# Impact of temperature on mortality in Hubei, China: a multi-county time series analysis

**DOI:** 10.1038/srep45093

**Published:** 2017-03-22

**Authors:** Yunquan Zhang, Chuanhua Yu, Junzhe Bao, Xudong Li

**Affiliations:** 1 Department of Epidemiology and Biostatistics, School of Health Sciences, Wuhan University, 185 Donghu Road, Wuchang District, Wuhan 430071, China; 2 Global Health Institute, Wuhan University, 8 Donghunan Road, Wuchang District, Wuhan 430072, China; 3 Department of Health Policy and Management, School of Public Health, Sun Yat-sen University, 74 Zhongshan 2nd Road, Guangzhou 510080, China; 4 Guangzhou Key Laboratory of Environmental Pollution and Health Risk Assessment, School of Public Health, Sun Yat-sen University, 74 Zhongshan 2nd Road, Guangzhou 510080, China; 5 Office of Epidemiology, Chinese Center for Disease Control and Prevention, 155 Changbai Road, Changping District, Beijing 102206, China

## Abstract

We examined the impact of extreme temperatures on mortality in 12 counties across Hubei Province, central China, during 2009–2012. Quasi-Poisson generalized linear regression combined with distributed lag non-linear model was first applied to estimate county-specific relationship between temperature and mortality. A multivariable meta-analysis was then used to pool the estimates of county-specific mortality effects of extreme cold temperature (1st percentile) and hot temperature (99th percentile). An inverse J-shaped relationship was observed between temperature and mortality at the provincial level. Heat effect occurred immediately and persisted for 2–3 days, whereas cold effect was 1–2 days delayed and much longer lasting. Higher mortality risks were observed among females, the elderly aged over 75 years, persons dying outside the hospital and those with high education attainment, especially for cold effects. Our data revealed some slight differences in heat- and cold- related mortality effects on urban and rural residents. These findings may have important implications for developing locally-based preventive and intervention strategies to reduce temperature-related mortality, especially for those susceptible subpopulations. Also, urbanization should be considered as a potential influence factor when evaluating temperature-mortality association in future researches.

Climate change has broad direct or indirect impacts on the vast majority of the world’s population, thus it is widely considered as the greatest global threat for human health in the 21st century^1^. An increased frequency and intensity of temperature extremes were projected to proceed worldwide, and unstable weather patterns will continue to occur, at least for the foreseeable future^2^,^3^. It was estimated that 7.71% (95% CI: 7.43–7.91) of daily total deaths were attributable to high and low ambient temperature in a recent multi-country observational study^4^. Therefore, it has become one of the most urgent tasks for governments and policy-makers to take efficient actions, in order to reduce the mortality burden of extreme temperatures on public health.

Temperature-mortality relationships have been described as U-, V-, W, or J-shaped in numerous previous epidemiological studies^5^,^6^,^7^,^8^,^9^,^10^, which revealed the adverse health impacts of low and/or high temperatures. However, temperature-related mortality effects varied greatly by countries and regions, and differed even from city to city^4^,^7^,^11^,^12^. Moreover, climate patterns, socioeconomic status (e.g., education attainment), and study populations (e.g., different genders and age groups) can also modify temperature-mortality associations^13^,^14^,^15^.

Studies investigating temperature–mortality associations started later in mainland China, compared with those conducted in high-income countries/regions. In recent years, mortality impact assessment of extreme temperatures has gotten increasing attention in China. Furthermore, a number of multi-city studies have been conducted across a large span of latitudes and various climate characteristics^6^,^8^,^12^,^16^,^17^. Nevertheless, these multi-city studies mainly focused on some major metropolises, such as provincial capital cities or direct-controlled municipalities. In spite of less researches reported in rural counties, rural populations may exhibit different patterns of vulnerability to cold and heat effects^18^,^19^, considering the huge differences of living conditions and healthcare systems between urban and rural areas.

A Chinese national analysis divided the selected 66 communities into northeast, north, northwest, east, central, southwest, and south China, and demonstrated distinct temperature-mortality relationships in the seven geographical regions^20^. It was thus inferential that, in such a large country like China with very imbalanced economic and social development, regional multi-center studies provided more targeted policy support for developing local adaptive strategies and preventive warning system of temperature extremes.

In this study, we conducted a provincial analysis from 12 urban and rural counties across Hubei Province in China, to assess the impact of extreme temperatures on mortality, and explore the potential differences in cold- and heat- related mortality effects between urban and rural counties at the provincial level. The findings may contribute to developing effective adaptation and intervention strategies in response to climate change for local government and the public in Hubei, China.

## Results

Table S1 describes county-specific population characteristics by gender and age group for the 12 counties across Hubei Province in China. The population sizes of the selected counties in 2010 ranged from 0.15 million (Wujiagang) to 1.63 million (Tianmen). Table 1 gives the descriptive statistics on population size, daily mortality and weather conditions. A total of 146,676 non-accidental deaths were included in this study and a daily average of 9.1 deaths (from 2.3 to 22.2) were observed. The 12 counties showed similar climate characteristics. The annual mean temperature was 16.6 °C, with the lowest in Wufeng (14.7 °C) and the highest in Huangshigang (17.4 °C). The daily mean relative humidity was 74.7% and ranged from 67.6% in Macheng to 77.1% in Huangshigang.

**Figure 1:**
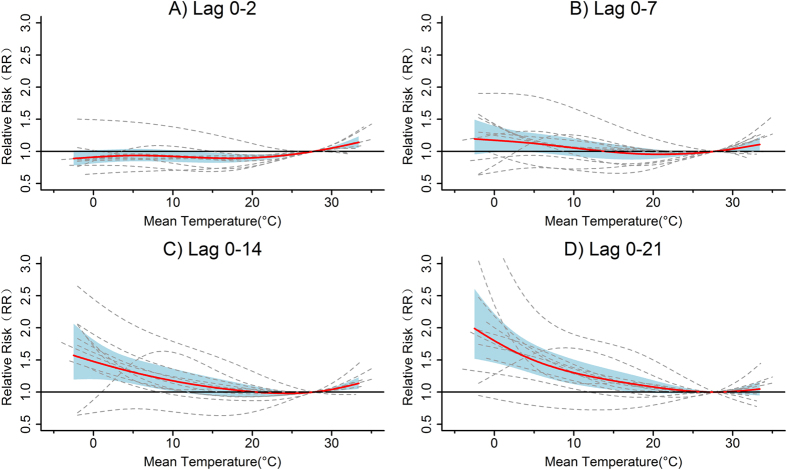
County-specific and pooled temperature-mortality relationships at lag 0–2 (**A**), lag 0–7 (**B**), lag 0–14 (**C**) and lag 0–21 (**D**). The continuous bold red lines represent the pooled curves and the blue areas are the 95% confidential intervals, whereas the long-dashed grey lines are the county-specific estimates. The reference temperature was 27.7 °C.

Figure 1 shows the county-specific and pooled exposure-response curves between mean temperature and daily mortality at different cumulative lag days. In general, the 12 counties across Hubei Province exhibited similar county-specific temperature-mortality relationships, and the pooled non-linear curves were found to be J-shaped or inverse J-shaped. For lag 0–2, high temperatures significantly increased mortality risk to some extent, whereas cold temperatures presented a certain protective effect. Growing adverse mortality impacts of cold temperatures were consistently observed when extending cumulative lag days from 0–7 to 0–21. Nevertheless, the effects of high temperatures weakened gradually and showed little influence in increasing mortality risk at lag 0–21 days.

**Figure 2:**
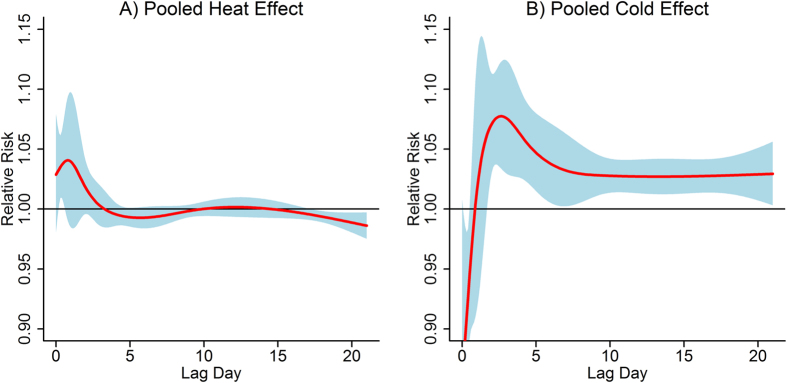
Lag patterns for pooled heat effect and cold effect on non-accidental mortality of the 12 counties in Hubei Province. The bold red lines are the effect estimates and the blue areas represent the 95% confidential intervals.

Figure 2 illustrates the lag patterns for pooled heat effect and cold effect on non-accidental mortality of the 12 counties in Hubei Province. Heat effect occurred immediately and persisted for 2–3 days, and some potential mortality displacement was observed in longer lags; whereas cold effect was 1–2 days delayed and much longer lasting. Similar lag patterns were also observed among subgroups stratified by gender and age group (Figures S1 and 2).

Figure S3 shows county-specific and pooled cold effects, which were much larger than heat effects (Figure S4). Pooled mortality risk was 1.097 (95% CI: 1.044–1.153) for heat effect at lag 0–2 days, and 1.828 (1.468–2.277) for cold effect at lag 0–21 days. Both county-specific heat and cold effects showed some heterogeneity. Heat effects varied from 0.965 (0.852, 1.093) in Gucheng to 1.243 (1.119–1.381) in Tianmen (Figure S4), while cold effects were found to be the lowest with 0.874 (0.469–1.629) in Gucheng and the highest with 3.387 (1.922–5.970) in Yunmeng (Figure S3).

Table 2 summarizes the pooled mortality risks of heat and cold effects at lag 0–2 days and lag 0–21 days, stratified by gender, age, education attainment, and place of death. Effect differences between subgroups were more obviously observed in cold effects rather than heat effects. Both heat and cold effects were significantly associated with increased mortality risks among females and males, while the effect estimates were slightly higher among females. Compared with those less than 75 years old, the elderly aged over 75 years showed a relatively higher mortality risk for heat effect and a distinctly higher mortality risk for cold effect. More pronounced and higher mortality effects were observed for those dying outside the hospital than those dying in hospital. Those with higher education attainment, instead of those with low education attainment, were found to be significantly affected by heat and cold effects.

**Figure 3:**
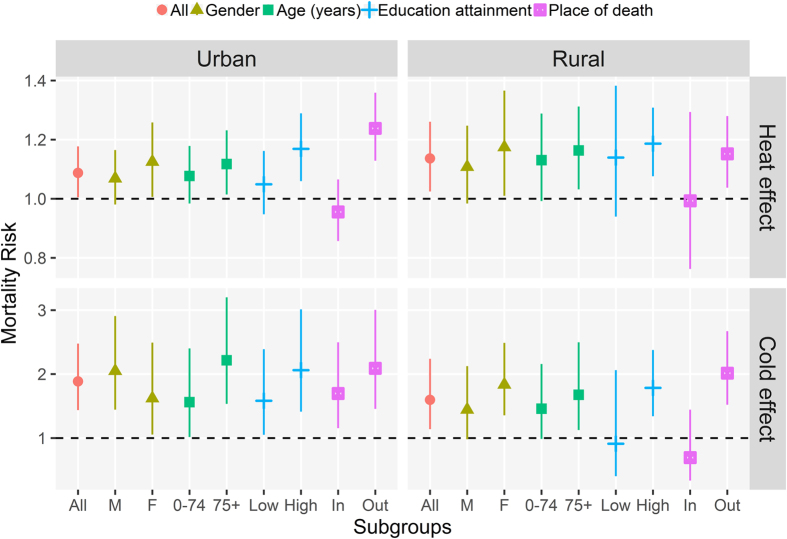
Pooled mortality risks and their 95% confidential intervals of heat effect (lag 0–2) and cold effect (lag 0–21) for urban and rural counties across Hubei Province in China, stratified by gender, age, education attainment, and place of death.

Figure 3 calculates the pooled mortality risks of heat effect (lag 0–2) and cold effect (lag 0–21) for urban and rural counties. Generally, both urban and rural counties showed consistent results with those subgroup analyses presented in Table 2, whereas we also found some differences in effect estimates. Heat effects were slightly higher in rural counties, while stronger cold-related mortality impacts were found in urban counties. A substantial difference among subgroups stratified by place of death was observed for heat effect in urban counties while for cold effect in rural counties.

## Discussion

In this epidemiologic investigation, we examined temperature-mortality relationships using consistent statistical methods in 12 counties across Hubei Province of central China during 2009–2012. The analysis identified distinct exposure-response and lag-response patterns at the provincial level in mortality effects of high and low temperatures, which may differ greatly in subgroups stratified by some individual factors, such as age, education attainment and place of death. Further, our data revealed some slight differences in heat- and cold- related mortality effects on urban and rural residents. These findings may have important implications for public health policies in Hubei Province, China.

Based on the 12 selected counties included in our study, we found an overall inverse J-shaped association between mean temperature and mortality at the provincial level. However, temperature-mortality associations varied greatly from a worldwide perspective^4^,^7^,^21^,^22^. As demonstrated in many epidemiologic investigations, these huge heterogeneity in health effects of temperature can be attributed a lot to geographical locations (e.g., latitudes) and climate features^14^,^23^,^24^,^25^. Consistent with another regional analysis of 9 communities in central China^20^, the present study revealed that, low temperatures rather than high temperatures did significantly increase mortality risks at lag 0–21 days, and comparable mortality risks were further observed when estimating pooled cold and heat effects.

As shown by a global analysis using data from 384 locations in 13 countries and regions^4^, far more temperature-attributable deaths were caused by cold (7.29%, 7.02–7.49) than by heat (0.42%, 0.39–0.44). Further more, a national investigation conducted in 16 large Chinese cities during 2007–2013 found that, cold temperatures accounted for about 92.4% (15.8% out of 17.1%) and 90.3% (13.1% out of 14.5%) of total cardiovascular and stroke deaths attributable to non-optimum temperatures, respectively^11^,^12^. These large-scale studies confirmed our findings that cold-related mortality impact contributed to the majority of health burden due to non-optimum temperatures.

Consistent with most previous findings, our study demonstrated that heat effects appeared immediately and usually lasted only 2 or 3 days, whereas cold effects were 1–2 days delayed but more long-lasting. In our analyses assessing mortality effect of temperature, 21 days was determined as the maximum lags, since using short lags cannot completely capture the effects of both low and high temperatures^10^. Cold effects could be underestimated because they usually lasted more than a week, while heat effects may be overestimated because potential mortality displacement might occur in longer lags^9^,^10^. In our results there was evidence of some mortality displacement, which could be observed during lags of 4–10 days and 15 days later. This phenomenon of harvest effect was considered to explain at least some of the observed mortality effect of high temperatures for the present study^26^. However, the presence of displacement phenomenon for heat effects varied greatly in different countries and regions^7^. It could be thus inferred that, only timely preventive measures do help in weakening the health impacts of high temperatures, while several weeks’ protection should be implemented to reduce the mortality burden from low temperatures^7^.

In this study, several potential individual-level modifiers were identified in cold- and heat-related mortality impacts, and the differences of these effect estimates between subgroups mainly focused on the effects of cold temperatures (Table 2). We found stronger associations among females than males, older people than young people, and residents dying outside the hospital than those dying in hospital. These findings were in accordance with most previous studies^27^,^28^,^29^,^30^. Due to the differences in physiological functions and health status, thermoregulatory and adaptive capacity, and social and living conditions, females and the elderly usually have greater susceptibility to temperature extremes^18^,^31^. Since individual-level older ages and community-level percentage of elderly people were positively associated with increased mortality effects of extreme temperatures^14^,^32^,^33^, and further considering the rapid aging trend of Chinese society, a growing mortality burden will be attributable to extreme temperatures among Chinese population in the coming decades^6^,^15^,^32^. Residents dying outside the hospital had higher mortality risk, the finding of which could be biologically explained by higher intensity of exposure to a cold and heat environment, worse healthcare and health insurance system for those dying outside the hospital^14^. The fact of social inequality calls for more attention in China, and central and local governments should make great efforts to improve the healthcare provision, especially for the social disadvantaged group^15^.

Education attainment is regarded as one of the most important factors reflecting one’s overall socioeconomic status. A number of previous studies^28^,^34^,^35^,^36^ revealed that, persons with lower education attainment were more vulnerable to temperature-related mortality, which could partly result from poorer housing conditions and health status, and more limited access to health care. Surprisingly, our investigation showed that those with high education level suffered more greatly to temperature-related mortality. This contrary result, which may seem irrational to some researchers, was also observed in another two recent multi-city studies conducted in China^15^,^37^. There are several possible explanations for these inconsistent results. Firstly, higher educated groups are expected to have longer lifespans^38^. In other words, those with higher education attainment generally die at relatively older ages (Table S2). This would increase the proportion of higher educated groups in the elderly (Table S2), who are more vulnerable to temperature extremes. Secondly, highly educated population are mostly brainworkers and generally of poor adaptability to temperature changes due to relative lack of physical exercise^37^. Besides, the obtained data on education attainment for those deaths were of rough stratification (e.g., 0–6, 7+ years and unclear) containing some missing information, and this incompleteness in collected data may result in some unavoidable bias^37^ and have limited our ability to detect the tenuous difference in the present study. Therefore, further well-designed large-scale investigations should be conducted to better understand the underlying reason for this discrepancy.

Previously, persons living in urban counties were identified as more vulnerable to extreme heat, which was attributed to the urban heat island effect and high population density^15^. Nevertheless, rural counties usually have poor infrastructure for heatstroke prevention and cooling system in summer (e.g., air conditioning usage), as well as pool knowledge and behavior coping with high temperatures^19^. Our study showed stronger heat effects in rural counties than in urban counties, which was in line with a previous study^39^. Moreover, a recent provincial investigation conducted in Jiangsu, China found that, less urban counties showed a higher pooled heat-related mortality risk with 1.43 (95% Posterior Intervals: 1.36–1.50) compared with 1.26 (1.23–1.30) in more urban counties^33^. These inconsistent results highlight a need to comprehensively understand the contributing proportion attributable to those independent factors influencing heat-related mortality, in order to design county-targeted preventive and control strategies. For cold effects, we observed more significant differences in rural counties among subgroups stratified by place of death. Compared with urban counties, rural counties in Hubei, China have several times broader area, and road transport in the vast majority of remote countrysides was hampered by mountains and hills, hence resulting in greater inequality in accessing timely emergency and treatment services during extreme cold temperatures. Besides, due to the influence of the Chinese tradition, rural residents who are almost dying tend to stay at home rather than go to hospital, which may be an additional reason for more deaths occurring outside the hospital in rural counties^18^.

Several limitations of this study should be noted. Firstly, we did not adjust for air pollution in the analysis due to data unavailability. A number of recent investigations have revealed that both cold- and heat-related mortality risks kept almost unchanged with and without air pollutants being controlled^7^,^30^,^40^. Thus, our main results are not expected to be significantly affected. Secondly, the meteorological variables came from only one basic-reference surface weather observation station or automatic station in a county, which may induce some degree of exposure misclassification^27^. However, given the fact that the weather data would not vary substantially within a county with a relative small geographic area, these station-based environmental monitoring factors would be able to basically represent the local weather pattern^20^,^27^. Finally, since some county-specific socioeconomic indicators (e.g., per capita income, afforestation coverage ratio, and ownership number of air-conditioning per 100 households) were not available in this study, and also limited by the relative small number of included counties, we could not further explore potential county-level effect modifiers of the temperature-mortality relationship^14^,^33^, which would be helpful in developing more effective intervention strategies to reduce cold- and heat-related mortality burden.

## Conclusions

This study suggested that short-term exposures to both low and high temperatures were associated with increased mortality in Hubei, China, while the cold effect was more pronounced and long-lasting than the heat effect. Females, the elderly, and those with high education attainment and dying outside the hospital were more vulnerable to extreme temperatures. Also, urbanization should be considered as a potential influence factor when evaluating temperature-mortality association in future epidemiological studies. These findings may have important implications for developing locally-based preventive and intervention strategies to reduce temperature-related mortality, especially for those susceptible subpopulations.

## Materials and Methods

### Study area and population

**Figure 4:**
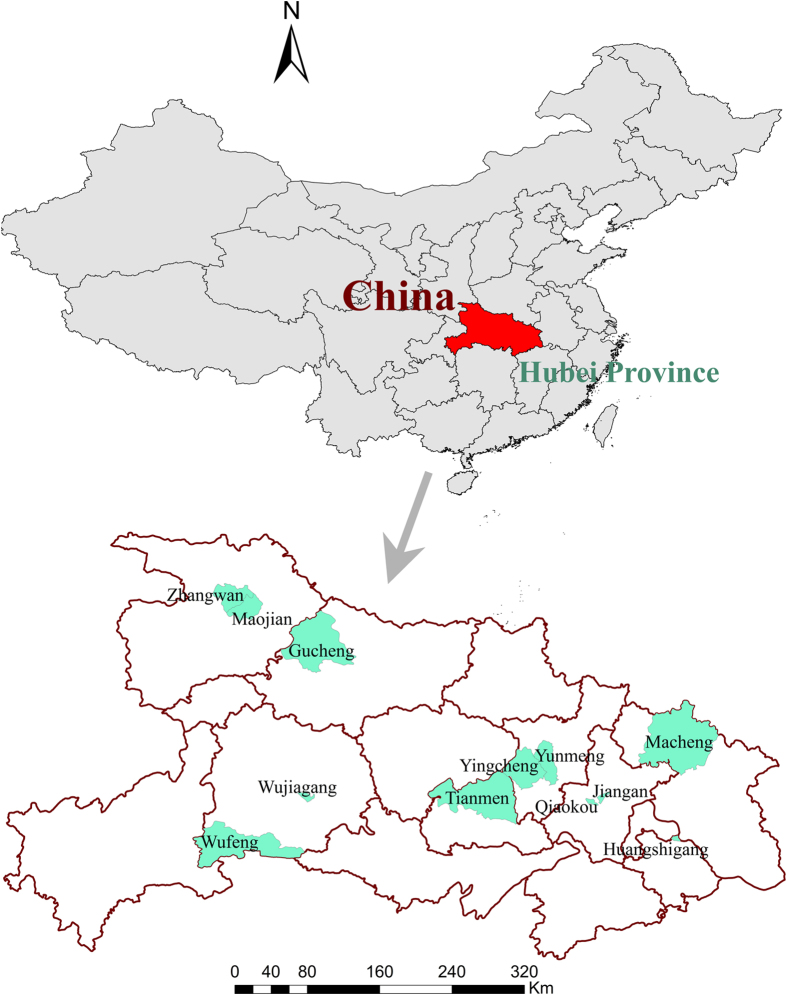
Locations of the 12 selected counties in Hubei Province, China. The maps were created using ArcGIS (Version 10.2, ESRI, http://www.esri.com/).

The National Disease Surveillance Points (DSPs) System of China established in 1980 was administrated by the Chinese Center for Disease Control and Prevention (China CDC). The system during the study period consisted of 161 nationally representative urban and rural sites from 31 provinces covering 78 million people, roughly 6% of Chinese population^41^. Located in central China and the middle reaches of the Yangtze River, Hubei Province had a population of 58.16 million in 2010 and covered an area of 185.9 thousands km^2^. DSPs distributed across Hubei Province included twelve counties, which were selected as the provincially representative sample in the present study^42^ (Fig. 4). Six counties including Jiangan, Qiaokou, Huangshigang, Zhangwan, Maojian and Wujiagang were urban communities (districts), and others were rural communities (villages or countrysides). The 12 selected counties are home to 6.7 million inhabitants, which accounts for 11.6% of the total population in Hubei Province.

### Data collection

County-specific daily non-accidental mortality data from January 1, 2009 through December 31, 2012 were obtained from the National Death Registration Reporting Information System of DSPs. Generally, for every death case, the Medical Certificate of Cause of Death must be completed in a very standardized form by the hospital or community/village doctor with the practicing physician qualification. The death information can be then registered into the network reporting system of the local CDC. Non-accidental causes of deaths were categorized using codes A00–R99 from the International Classification of Diseases 10th Revision (ICD–10). Daily non-accidental deaths were also divided into several subgroups stratified by gender, age (0–74 years, 75+ years), education attainment (low: 0–6 years, high: 7+ years), and place of death (in hospital or outside the hospital).

County-specific daily meteorological data on maximum, mean, and minimum temperature and relative humidity for the same period were obtained from the China Meteorological Data Sharing Service System (http://cdc.cma.gov.cn). Meteorological data for each community were obtained from one basic-reference surface weather observation station or automatic station. Limited by data availability and based on the assumption that weather data would not vary substantially within a city, we used meteorological data of Wuhan City for two of her urban districts (Jiangan and Qiaokou), and Xiaogan City for two of her rural counties (Yingcheng and Yunmeng).

### Data analysis

A two stage approach was used to conduct data analysis in the present study, which was well documented in previous studies^25^. In the first stage, we applied a standard time-series quasi-Poisson generalized linear model (GLM) combined with distributed lag nonlinear model (DLNM) to estimate county-specific association between temperature and mortality^43^,^44^. Briefly, the GLM regression included the following covariates: (1) a natural cubic smooth function of time with 7 degrees of freedom (df) per year to control for long-term and seasonal trends; (2) a natural cubic smooth function of relative humidity with 3 df; and (3) indicator variables for “day of the week (DOW)” and public holiday. DLNM was then used to model the exposure-response association with a natural cubic spline with three internal knots placed at the 10th–50th–90th percentiles of county-specific temperature distributions, and the lag-response association with a natural cubic spline and knots placed at equally-spaced values in the log scale^4^,^20^. To completely capture the overall effect of cold temperatures and adjust for any potential harvesting for hot temperatures, we used 21 days as the maximum lag for temperature according to previous studies^4^,^7^,^20^.

The above county-specific association was then reduced to the overall temperature–mortality association, which cumulated the risk during the lag period (lag 0–21). Cumulative temperature–mortality associations for different days of lag, such as lag 0–2, lag 0–7 and lag 0–14, can also be obtained by conducting similar procedures.

In the second stage, we conducted a multivariate meta-analytical model^45^ to pool the estimated county-specific cumulative temperature–mortality relationship for different days of lag so as to examine the exposure-response association at the provincial level. To quantitatively estimate the mortality risks of exposure to cold and hot temperatures, we first extracted the minimum-mortality temperature (MMT) based on the county-specific overall temperature–mortality association. And county-specific MMT was then used as the reference temperature to calculate the mortality risks at 1st percentile (cold temperature) and 99th percentile (hot temperature) of mean temperature distribution, which corresponded to county-specific cold effect and heat effect, respectively^20^. Pooled lag-response associations for cold and heat effects can be also obtained by conducting additional multivariable meta-analysis described above.

To check the main findings of this study, sensitivity analyses were performed by changing df (3–6 per year) in the smoothness of time. We also changed df (4–6) for humidity, and varied the maximum lags from 22 to 28 days in the temperature-DLNMs.

The statistical tests were two-sided, and effects of *p* < 0.05 were considered statistically significant. All the analyses were performed with the R software (version 3.2.2; http://www.r-project.org/). County-specific temperature–mortality associations were estimated using R package “dlnm”. Multivariate meta-analysis was conducted using package “mvmeta”.

## Additional Information

**How to cite this article:** Zhang, Y. *et al*. Impact of temperature on mortality in Hubei, China: a multi-county time series analysis. *Sci. Rep.* **7**, 45093; doi: 10.1038/srep45093 (2017).

**Publisher's note:** Springer Nature remains neutral with regard to jurisdictional claims in published maps and institutional affiliations.

## Supplementary Material

Supplementary Information

## Figures and Tables

**Table 1:** Descriptive statistics for county-specific population size, daily mortality, mean temperature and relative humidity for the 12 counties across Hubei Province in China, 2009–2012.

County	Population size (million)	Non-accidental death		Mean temperature (°C)						Relative humidity (%)
		Total	Mean ± SD	Mean ± SD	P_1_	P_25_	P_50_	P_75_	P_99_	
Urban										
Jiangan	0.68	16,895	11.6 ± 3.9	16.8 ± 9.6	−0.6	8.2	18.1	25.0	32.8	76.6
Qiaokou	0.54	14,362	9.8 ± 3.6	16.8 ± 9.6	−0.6	8.2	18.1	25.0	32.8	76.6
Huangshigang	0.17	3,496	2.4 ± 1.6	17.4 ± 9.3	0.1	8.9	18.9	25.4	33.0	77.1
Zhangwan and Maojian	0.39	7,805	5.3 ± 2.8	15.4 ± 9.0	−0.8	7.0	16.6	23.0	30.6	74.8
Wujiagang	0.15	3,382	2.3 ± 1.6	17.2 ± 8.8	1.0	9.2	18.2	24.8	31.9	74.4
Rural										
Wufeng	0.21	4,475	3.1 ± 1.9	14.7 ± 8.2	−0.7	7.5	15.3	22.0	28.2	76.2
Macheng	1.18	24,980	17.1 ± 7.4	17.1 ± 9.6	−0.4	8.5	18.6	25.3	33.0	67.6
Gucheng	0.54	12,899	8.8 ± 4.3	16.2 ± 9.3	−0.9	7.7	17.8	24.2	30.9	73.2
Yingcheng	0.64	13,496	9.2 ± 3.6	16.5 ± 9.5	−0.7	7.9	17.9	24.7	32.0	76.6
Yunmeng	0.56	12,427	8.5 ± 3.8	16.5 ± 9.5	−0.7	7.9	17.9	24.7	32.0	76.6
Tianmen	1.63	32,459	22.2 ± 7.3	17.1 ± 9.3	−0.3	8.6	18.2	25.2	32.6	72.5
Overall	6.70	146,676	9.1 ± 7.3	16.6 ± 9.3	−0.5	8.2	17.7	24.4	32.4	74.7

**Table 2:** Pooled mortality risks and their 95% confidential intervals of heat and cold effects at lag 0–2 days and lag 0–21 days in 12 counties across Hubei Province in China, stratified by gender, age, education attainment, and place of death.

Subgroups	Heat effect		Cold effect	
	Lag 0–2	Lag 0–21	Lag 0–2	Lag 0–21
All	**1.097 (1.044, 1.153)**	1.027 (0.960, 1.099)	0.907 (0.808, 1.017)	**1.828 (1.468, 2.277)**
Gender				
Male	**1.060 (1.009, 1.113)**	1.011 (0.941, 1.086)	0.917 (0.803, 1.047)	**1.767 (1.377, 2.268)**
Female	**1.134 (1.052, 1.221)**	1.043 (0.938, 1.160)	0.897 (0.773, 1.040)	**1.910 (1.464, 2.494)**
Age (years)				
0–74	**1.061 (1.008, 1.117)**	1.011 (0.932, 1.097)	0.914 (0.798, 1.046)	**1.489 (1.134, 1.956)**
75+	**1.164 (1.087, 1.247)**	1.066 (0.944, 1.203)	0.939 (0.815, 1.081)	**2.241 (1.735, 2.894)**
Education attainment (years)				
Low (0–6)	1.069 (0.991, 1.153)	1.079 (0.902, 1.291)	0.918 (0.775, 1.087)	1.286 (0.888, 1.862)
High (7+)	**1.134 (1.080, 1.191)**	1.019 (0.950, 1.092)	0.918 (0.803, 1.050)	**2.028 (1.599, 2.571)**
Place of death				
In the hospital	0.991 (0.904, 1.087)	1.076 (0.911, 1.271)	0.781 (0.612, 0.995)	1.259 (0.854, 1.857)
Outside the hospital	**1.156 (1.086, 1.232)**	0.978 (0.887, 1.078)	0.949 (0.804, 1.121)	**2.086 (1.638, 2.656)**

## References

[b1] Anthony C. . Managing the health effects of climate change: Lancet and University College London Institute for Global Health Commission. Lancet 373, 1693–1733 (2009). 19447250 10.1016/S0140-6736(09)60935-1

[b2] Semenza J. C. Climate change and human health. Int J Environ Res Public Health 11, 7347–7353, doi: 10.3390/ijerph110707347 (2014). 25046633 PMC4113880

[b3] Guo Y. . Projecting future temperature-related mortality in three largest Australian cities. Environ Pollut 208, 66–73, doi: 10.1016/j.envpol.2015.09.041 (2016). 26475058

[b4] Gasparrini A. . Mortality risk attributable to high and low ambient temperature: a multicountry observational study. Lancet 386, 369–375, doi: 10.1016/S0140-6736(14)62114-0 (2015). 26003380 PMC4521077

[b5] Onozuka D. & Hagihara A. Variation in vulnerability to extreme-temperature-related mortality in Japan: A 40-year time-series analysis. Environ Res 140, 177–184, doi: 10.1016/j.envres.2015.03.031 (2015). 25863590

[b6] Ma W., Chen R. & Kan H. Temperature-related mortality in 17 large Chinese cities: how heat and cold affect mortality in China. Environ Res 134, 127–133, doi: 10.1016/j.envres.2014.07.007 (2014). 25127523

[b7] Guo Y. . Global variation in the effects of ambient temperature on mortality: a systematic evaluation. Epidemiology 25, 781–789, doi: 10.1097/EDE.0000000000000165 (2014). 25166878 PMC4180721

[b8] Bao J., Wang Z., Yu C. & Li X. The influence of temperature on mortality and its Lag effect: a study in four Chinese cities with different latitudes. BMC Public Health 16, 375, doi: 10.1186/s12889-016-3031-z (2016). 27146378 PMC4855424

[b9] Zhang Y. . The Short-Term Effect of Ambient Temperature on Mortality in Wuhan, China: A Time-Series Study Using a Distributed Lag Non-Linear Model. Int J Environ Res Public Health 13, 722, doi: 10.3390/ijerph13070722 (2016). PMC4962263 27438847

[b10] Guo Y., Barnett A. G., Pan X., Yu W. & Tong S. The impact of temperature on mortality in Tianjin, China: a case-crossover design with a distributed lag nonlinear model. Environ Health Perspect 119, 1719–1725, doi: 10.1289/ehp.1103598 (2011). 21827978 PMC3261984

[b11] Yang J. . The burden of stroke mortality attributable to cold and hot ambient temperatures: Epidemiological evidence from China. Environ Int 92–93, 232–238, doi: 10.1016/j.envint.2016.04.001 (2016). 27107228

[b12] Yang J. . Cardiovascular mortality risk attributable to ambient temperature in China. Heart 101, 1966–1972, doi: 10.1136/heartjnl-2015-308062 (2015). 26567233

[b13] Medina-Ramon M. & Schwartz J. Temperature, temperature extremes, and mortality: a study of acclimatisation and effect modification in 50 US cities. Occup Environ Med 64, 827–833, doi: 10.1136/oem.2007.033175 (2007). 17600037 PMC2095353

[b14] Huang Z. . Individual-level and community-level effect modifiers of the temperature-mortality relationship in 66 Chinese communities. BMJ Open 5, e009172, doi: 10.1136/bmjopen-2015-009172 (2015). PMC4577931 26369803

[b15] Ma W. . The short-term effect of heat waves on mortality and its modifiers in China: An analysis from 66 communities. Environ Int 75, 103–109, doi: 10.1016/j.envint.2014.11.004 (2015). 25461419

[b16] Guo Y. . Extremely cold and hot temperatures increase the risk of ischaemic heart disease mortality: epidemiological evidence from China. Heart 99, 195–203, doi: 10.1136/heartjnl-2012-302518 (2013). 23150195

[b17] Yang J. . The effect of ambient temperature on diabetes mortality in China: A multi-city time series study. Sci Total Environ 543, 75–82, doi: 10.1016/j.scitotenv.2015.11.014 (2016). 26580729

[b18] Zhou M. G. . Health impact of the 2008 cold spell on mortality in subtropical China: the climate and health impact national assessment study (CHINAs). Environ Health 13, 60, doi: 10.1186/1476-069X-13-60 (2014). 25060645 PMC4115219

[b19] Bai L., Woodward A., Cirendunzhu & Liu Q. County-level heat vulnerability of urban and rural residents in Tibet, China. Environ Health 15, 3, doi: 10.1186/s12940-015-0081-0 (2016). 26757705 PMC4711018

[b20] Ma W. . The temperature-mortality relationship in China: An analysis from 66 Chinese communities. Environ Res 137, 72–77, doi: 10.1016/j.envres.2014.11.016 (2015). 25490245

[b21] Gasparrini A. . Temporal Variation in Heat-Mortality Associations: A Multicountry Study. Environ Health Perspect 123, 1200–1207, doi: 10.1289/ehp.1409070 (2015). 25933359 PMC4629745

[b22] Gasparrini A. . Changes in Susceptibility to Heat During the Summer: A Multicountry Analysis. Am J Epidemiol 183, 1027–1036, doi: 10.1093/aje/kwv260 (2016). 27188948 PMC4887574

[b23] Anderson G. B. & Bell M. L. Heat waves in the United States: mortality risk during heat waves and effect modification by heat wave characteristics in 43 U.S. communities. Environ Health Perspect 119, 210–218, doi: 10.1289/ehp.1002313 (2011). 21084239 PMC3040608

[b24] Xiao J. . How much does latitude modify temperature-mortality relationship in 13 eastern US cities? Int J Biometeorol 59, 365–372, doi: 10.1007/s00484-014-0848-y (2015). 24880926

[b25] Gasparrini A. & Armstrong B. Reducing and meta-analysing estimates from distributed lag non-linear models. BMC Med Res Methodol 13, 1, doi: 10.1186/1471-2288-13-1 (2013). 23297754 PMC3599933

[b26] Basu R. & Malig B. High ambient temperature and mortality in California: exploring the roles of age, disease, and mortality displacement. Environ Res 111, 1286–1292, doi: 10.1016/j.envres.2011.09.006 (2011). 21981982

[b27] Tian Z., Li S., Zhang J., Jaakkola J. J. & Guo Y. Ambient temperature and coronary heart disease mortality in Beijing, China: a time series study. Environ Health 11, 56, doi: 10.1186/1476-069X-11-56 (2012). 22909034 PMC3490736

[b28] Son J. Y., Lee J. T., Anderson G. B. & Bell M. L. The impact of heat waves on mortality in seven major cities in Korea. Environ Health Perspect 120, 566–571, doi: 10.1289/ehp.1103759 (2012). 22266672 PMC3339449

[b29] Wu W. . Temperature-mortality relationship in four subtropical Chinese cities: a time-series study using a distributed lag non-linear model. Sci Total Environ 449, 355–362, doi: 10.1016/j.scitotenv.2013.01.090 (2013). 23454696

[b30] Chen K. . Influence of heat wave definitions to the added effect of heat waves on daily mortality in Nanjing, China. Sci Total Environ 506–507, 18–25, doi: 10.1016/j.scitotenv.2014.10.092 (2015). 25460935

[b31] Yu W. . Daily average temperature and mortality among the elderly: a meta-analysis and systematic review of epidemiological evidence. Int J Biometeorol 56, 569–581, doi: 10.1007/s00484-011-0497-3 (2012). 21975970

[b32] Li T. . Aging Will Amplify the Heat-related Mortality Risk under a Changing Climate: Projection for the Elderly in Beijing, China. Sci Rep 6, 28161, doi: 10.1038/srep28161 (2016). 27320724 PMC4913346

[b33] Chen K. . Urbanization Level and Vulnerability to Heat-Related Mortality in Jiangsu Province, China. Environ Health Perspect 124, 1863–1869, doi: 10.1289/EHP204 (2016). 27152420 PMC5132638

[b34] Yang J., Ou C. Q., Ding Y., Zhou Y. X. & Chen P. Y. Daily temperature and mortality: a study of distributed lag non-linear effect and effect modification in Guangzhou. Environ Health 11, 63, doi: 10.1186/1476-069X-11-63 (2012). 22974173 PMC3511876

[b35] O’Neill M. S. Modifiers of the Temperature and Mortality Association in Seven US Cities. American Journal of Epidemiology 157, 1074–1082, doi: 10.1093/aje/kwg096 (2003). 12796043

[b36] Curriero F. C. . Temperature and mortality in 11 cities of the eastern United States. Am J Epidemiol 155, 80–87 (2002). 11772788 10.1093/aje/155.1.80

[b37] Hu M. . Analysis of the impact of the Socio-economic factors on temperature-mortality association in southern China. Chinese Journal of Preventive Medicine 48, 401–405 (2014). 24985381

[b38] van Raalte A. A. . More variation in lifespan in lower educated groups: evidence from 10 European countries. International Journal of Epidemiology 40, 1703–1714, doi: 10.1093/ije/dyr146 (2011). 22268238

[b39] Sheridan S. C. & Dolney T. J. Heat, mortality, and level of urbanization: measuring vulnerability across Ohio, USA. Climate Res 24, 255–265 (2003).

[b40] Xie H. . Short-term effects of the 2008 cold spell on mortality in three subtropical cities in Guangdong Province, China. Environ Health Perspect 121, 210–216, doi: 10.1289/ehp.1104541 (2013). 23128031 PMC3569675

[b41] Zhou M., Jiang Y., Huang Z. & Wu F. Adjustment and representativeness evaluation of national disease surveillance points system. Disease Surveillance 25, 239–244 (2010).

[b42] Pan J. . Underreporting of death in disease surveillance system in Hubei, 2009–2011. Disease Surveillance 28, 478–480 (2013).

[b43] Gasparrini A., Armstrong B. & Kenward M. G. Distributed lag non-linear models. Stat Med 29, 2224–2234, doi: 10.1002/sim.3940 (2010). 20812303 PMC2998707

[b44] Gasparrini A. & Armstrong B. Time series analysis on the health effects of temperature: advancements and limitations. Environ Res 110, 633–638, doi: 10.1016/j.envres.2010.06.005 (2010). 20576259

[b45] Gasparrini A., Armstrong B. & Kenward M. G. Multivariate meta-analysis for non-linear and other multi-parameter associations. Stat Med 31, 3821–3839, doi: 10.1002/sim.5471 (2012). 22807043 PMC3546395

