# Supplementary material for: Impact of temperature on mortality in Hubei, China: a multi-county time series analysis
Source: Sci Rep. 2017 Mar 22;7:45093. doi: 10.1038/srep45093 (PMC5361185; doi:10.1038/srep45093)
Supplement: Supplementary Information [file srep45093-s1.pdf]

Supplementary Information

Impact of temperature on mortality in Hubei, China: a multi-county time series analysis

Yunquan Zhang [1], Chuanhua Yu [1, 2,*], Junzhe Bao [1, 3, 4], and Xudong Li [5]

1 Department of Epidemiology and Biostatistics, School of Health Sciences, Wuhan University, 185 Donghu Road,Wuchang District, Wuhan 430071, China;
2 Global Health Institute, Wuhan University, 8 Donghunan Road, Wuchang District, Wuhan 430072, China;
3 Department of Health Policy and Management, School of Public Health, Sun Yat-sen University, 74 Zhongshan 2nd Road, Guangzhou 510080, China;
4 Guangzhou Key Laboratory of Environmental Pollution and Health Risk Assessment, School of Public Health, Sun Yat-sen University, 74 Zhongshan 2nd Road, Guangzhou 510080, China;
5 Office of Epidemiology, Chinese Center for Disease Control and Prevention, 155 Changbai Road, Changping District, Beijing 102206, China.

***** Correspondence: YuCHua@whu.edu.cn (C.Y.); Tel. /Fax: +86-27-6875-9299.

Table S1. County-specific population characteristics by gender and age group for the 12 counties across Hubei Province in China, 2010. [a] Population number. [b] Population number (percentage accounting for total population).

County	Total population	Sex		Age (years)		
		Male [a]	Female [b]	0-44 [a]	45-74 [a]	75+ [b]
Urban						
Jiangan	683,030	340,480	342,550 (50.2)	393,907	258,039	31,084 (4.6)
Qiaokou	538,410	270,000	268,410 (49.9)	275,586	231,897	30,927 (5.7)
Huangshigang	174,002	87,331	86,671 (49.8)	105,076	62,634	6,292 (3.6)
Zhangwan and Maojian	392,592	201,700	190,892 (48.6)	230,060	152,154	10,378 (2.6)
Wujiagang	153,663	77,721	75,942 (49.4)	113,009	37,744	2,910 (1.9)
Rural						
Wufeng	210,430	109,684	100,746 (50.2)	146,691	57,768	5,971 (2.8)
Macheng	1,180,105	624,673	555,432 (49.9)	845,722	313,202	21,181 (1.8)
Gucheng	535,775	276,028	259,747 (49.8)	362,455	159,043	14,277 (2.7)
Yingcheng	636,473	333,245	303,228 (48.6)	441,483	182,191	12,799 (2.0)
Yunmeng	563,393	290,370	273,023 (49.4)	416,932	133,885	12,576 (2.2)
Tianmen	1,634,139	845,401	788,738 (48.8)	1,270,483	348,397	15,259 (0.9)
Overall	6,702,012	3,456,633	3,245,379 (48.8)	4,601,404	1,936,954	163,654 (2.4)

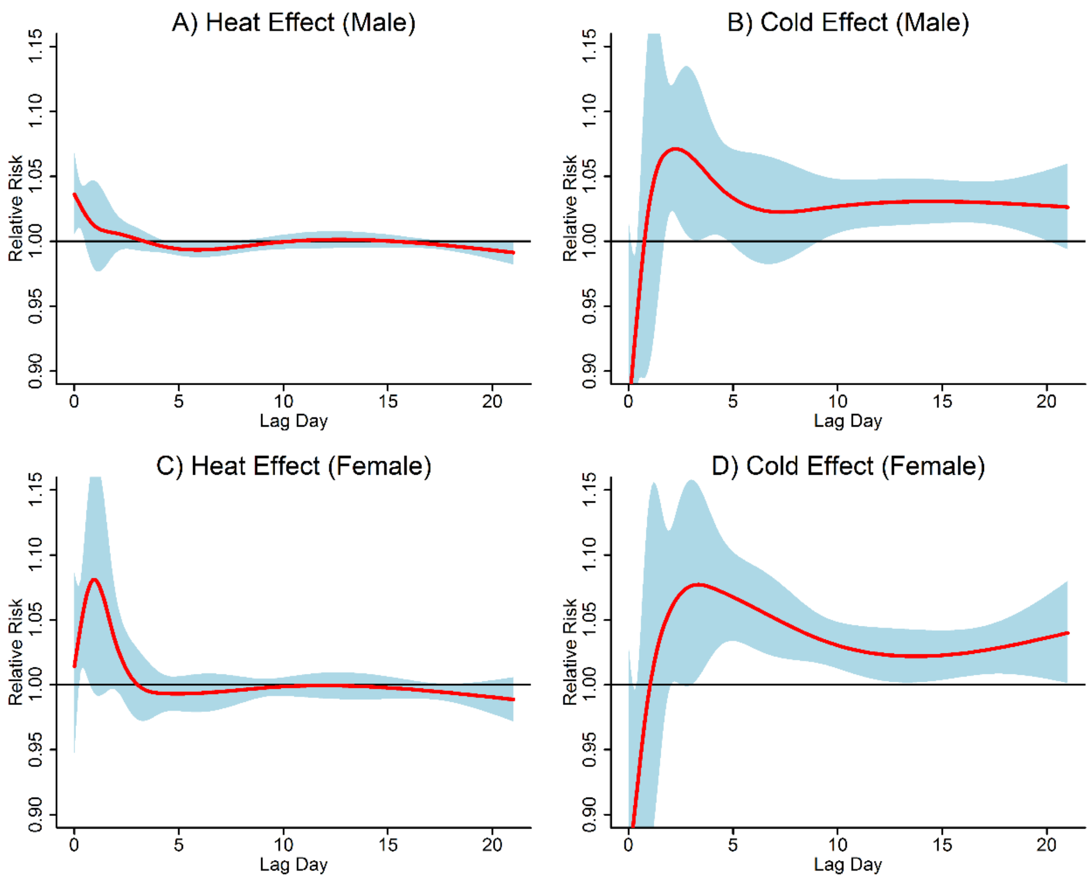

Figure S1. Lag patterns for pooled heat effect and cold effect, stratified by gender. The bold red lines are the effect estimates and the blue areas represent the 95% confidential intervals.

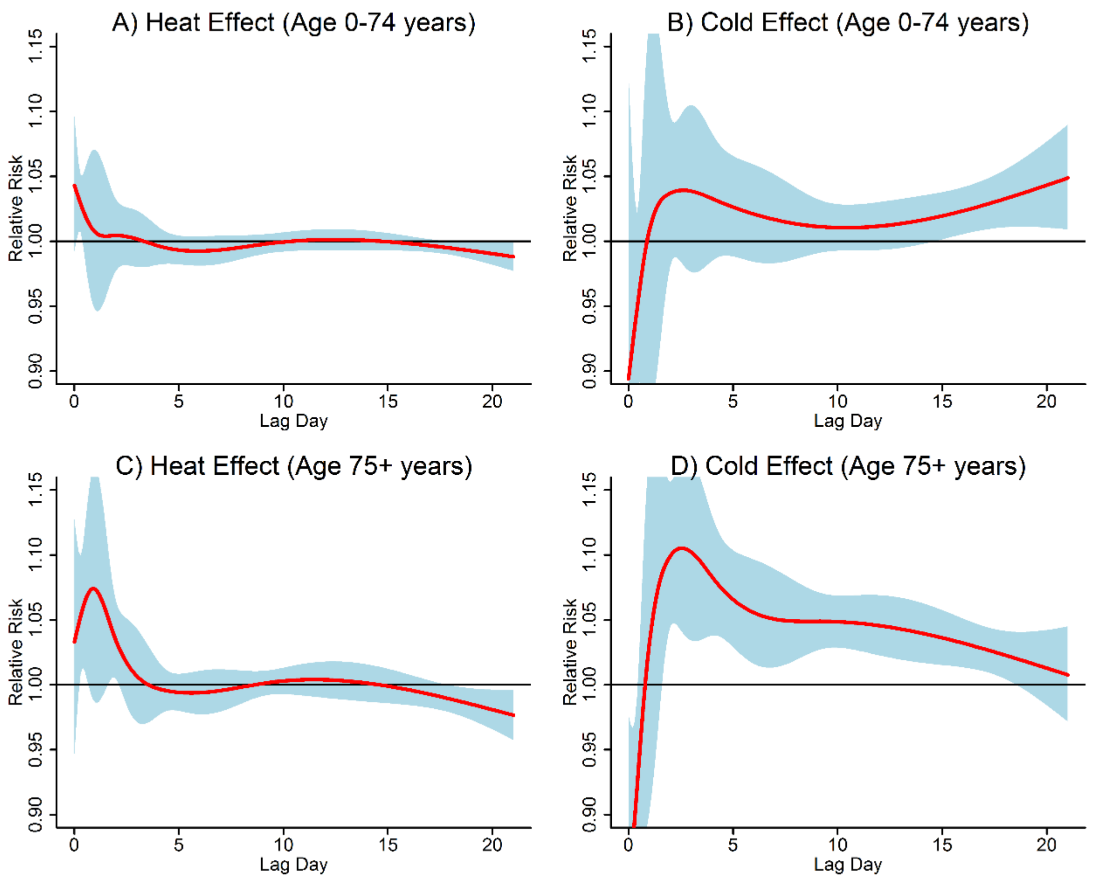

Figure S2. Lag patterns for pooled heat effect and cold effect, stratified by age group. The bold red lines are the effect estimates and the blue areas represent the 95% confidential intervals.

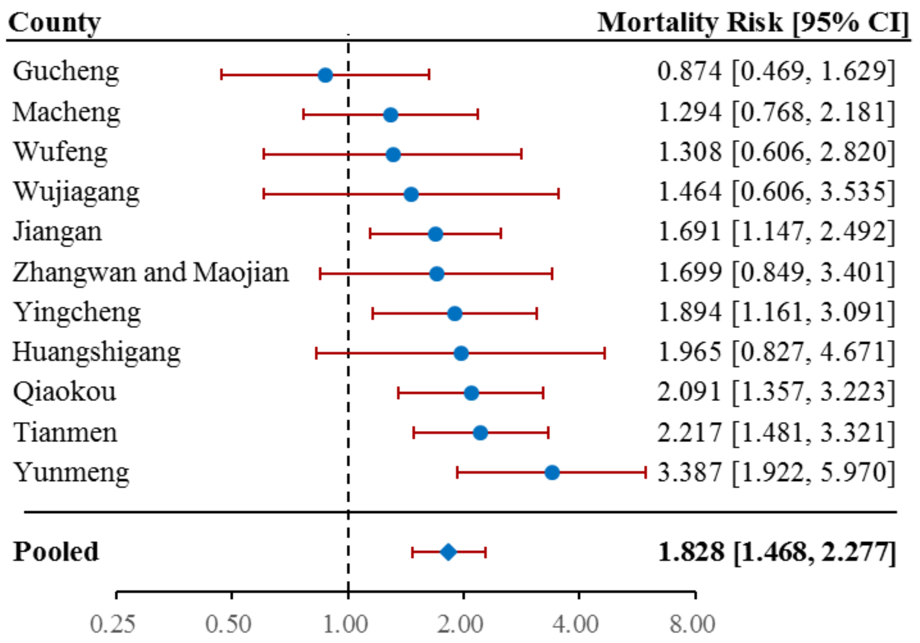

Figure S3. County-specific and pooled cold effects for lag 0-21 days. The blue dots represent the effect estimates and the horizontal red lines are the 95% confidential intervals.

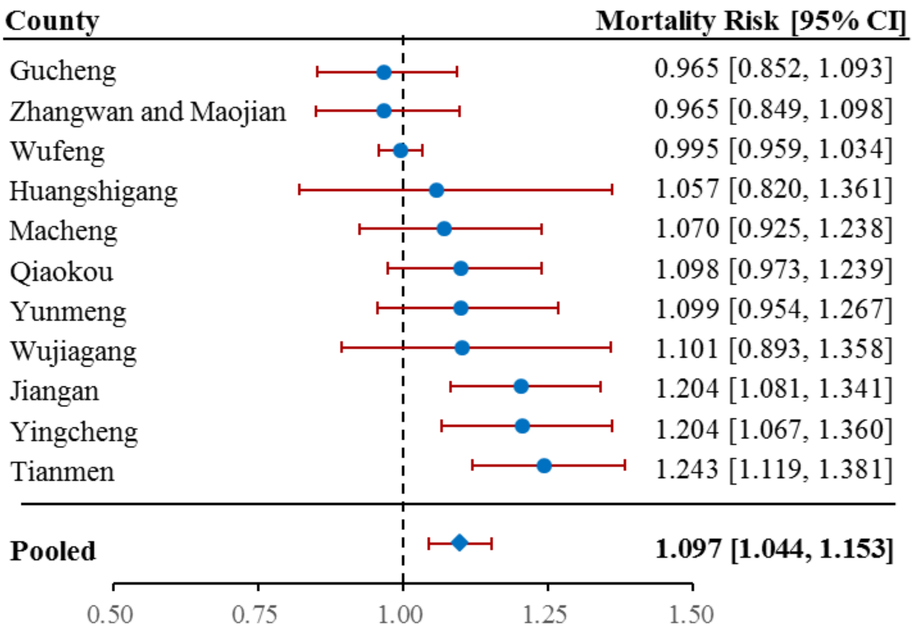

Figure S4. County-specific and pooled heat effects for lag 0-2 days. The blue dots represent the effect estimates and the horizontal red lines are the 95% confidential intervals.

Table S2. County-specific age characteristics of included non-accidental deaths stratified by education attainment for the 12 counties across Hubei Province in China, 2009-2012.

County	Percentage of education attainment unclear	High education attainment		Low education attainment	
		Mean age (years)	Percentage of 75+ years old (%)	Mean age (years)	Percentage of 75+ years old (%)
Urban					
Jiangan	1.8	79.1	78.6	67.6	41.0
Qiaokou	3.1	79.3	80.9	66.1	36.0
Huangshigang	5.1	76.2	66.7	64.2	31.5
Zhangwan and Maojian	11.7	70.4	47.8	60.6	24.2
Wujiagang	15.3	77.1	70.8	63.6	29.9
Rural					
Wufeng	2.3	73.9	59.9	54.6	20.1
Macheng	3.8	69.2	37.0	52.1	8.8
Gucheng	9.4	71.0	49.0	56.5	17.4
Yingcheng	4.4	73.4	56.1	54.0	16.1
Yunmeng	2.3	71.5	49.3	48.7	7.5
Tianmen	2.7	73.5	54.3	51.5	6.0
Overall	4.0	73.0	53.9	59.9	24.7
